# Unveiling the Hidden Culprit of Posterior Reversible Encephalopathy Syndrome Superimposing Postpartum Eclampsia: A Rare Case

**DOI:** 10.7759/cureus.57006

**Published:** 2024-03-26

**Authors:** Smruti A Mapari, Deepti Shrivastava, Gautam N Bedi, Utkarsh Pradeep

**Affiliations:** 1 Obstetrics and Gynecology, Jawaharlal Nehru Medical College, Datta Meghe Institute of Higher Education and Research, Wardha, IND; 2 Medicine, Jawaharlal Nehru Medical College, Datta Meghe Institute of Higher Education and Research, Wardha, IND

**Keywords:** encephalopathy, neurological disorder, eclampsia, postpartum, posterior reversible encephalopathy syndrome

## Abstract

The rare yet potentially fatal neurological complication known as posterior reversible encephalopathy syndrome (PRES) can manifest during pregnancy. Alongside symptoms such as headaches, nausea, visual disturbances, and altered mental status, patients often experience seizures or loss of consciousness. Imaging typically reveals vascular edema affecting the parietal and occipital lobes within the subcortical region. We present the case of a 24-year-old patient who developed postpartum eclampsia followed by PRES. MRI findings demonstrated hyperintensities in the posterior parietal, frontal, and occipital lobes bilaterally, confirming the diagnosis. Prompt administration of levetiracetam and labetalol led to the resolution of the patient’s symptoms. Subsequently, we thoroughly searched online databases for peer-reviewed articles examining the etiology, clinical presentation, and treatment options for PRES. Our evaluation of the case findings alongside existing literature underscored the rarity of PRES occurring concurrently with postpartum eclampsia, highlighting the importance of timely identification and intervention in managing this condition. Further research is warranted to enhance our understanding of PRES in the context of pregnancy-related complications.

## Introduction

A rare neurological condition known as posterior reversible encephalopathy syndrome (PRES) can affect pregnant women shortly after childbirth [1]. Various factors can contribute to PRES, including sepsis, autoimmune diseases, renal failure, and hypertensive encephalopathy [1,2]. Patients with PRES may present with non-specific symptoms such as headache, vomiting, visual abnormalities, and altered mental status [2]. Diagnosis typically relies on MRI, which reveals subcortical vasogenic edema in the bilateral occipital and parietal lobes [3].

Among the obstetric population, pre-eclampsia and eclampsia are the leading causes of PRES [4]. Pre-eclampsia, characterized by high blood pressure and increased proteinuria, can develop during pregnancy or in the postpartum period [5]. Eclampsia is diagnosed when seizures occur in the context of pre-eclampsia [5]. While pre-eclampsia and eclampsia typically manifest between 20 weeks of pregnancy and 48 hours postpartum, they can also arise up to four weeks postpartum [6]. Notably, fewer than 22% of PRES patients have a prior diagnosis of pre-eclampsia, often due to underreporting of symptoms [7]. This report highlights a case where eclampsia led to PRES without a history of pre-eclampsia. A literature review was conducted to better understand the pathophysiology, clinical manifestations, optimal diagnostic approach, treatment strategies, and potential side effects associated with PRES.

## Case presentation

A 24-year-old primigravida woman presented to the emergency department with a history of two episodes of seizures and a sudden onset of vision loss that had progressed over the past two days. She was on the third day of the postnatal period. The patient denied any history of bleeding, vomiting, or bowel complaints. She had no significant family or medical history and reported no episodes of headaches, visual disturbances, or altered mental status during her prenatal or intrapartum period. An ultrasound performed at 34 weeks gestation showed normal findings, and her discharge summary from a previous hospitalization indicated a lower-segment cesarean delivery due to polyhydramnios without any complications.

Upon admission, the patient was afebrile with a blood pressure of 150/100 mmHg and a pulse rate of 100 beats per minute. Bilateral pedal pitting edema was noted during the physical examination. She experienced a generalized tonic-clonic seizure later that day and complained of a headache with severe vision loss. Upon assessment by the medical team, she was found to be in a postictal state but was vitally stable with an SpO_2_ of 97%. Immediate interventions included securing the airway and administering intravenous (IV) 25% dextrose 100 mL, IV midazolam 2 mg, and IV 20% magnesium sulfate 4 g.

**Figure 1:**
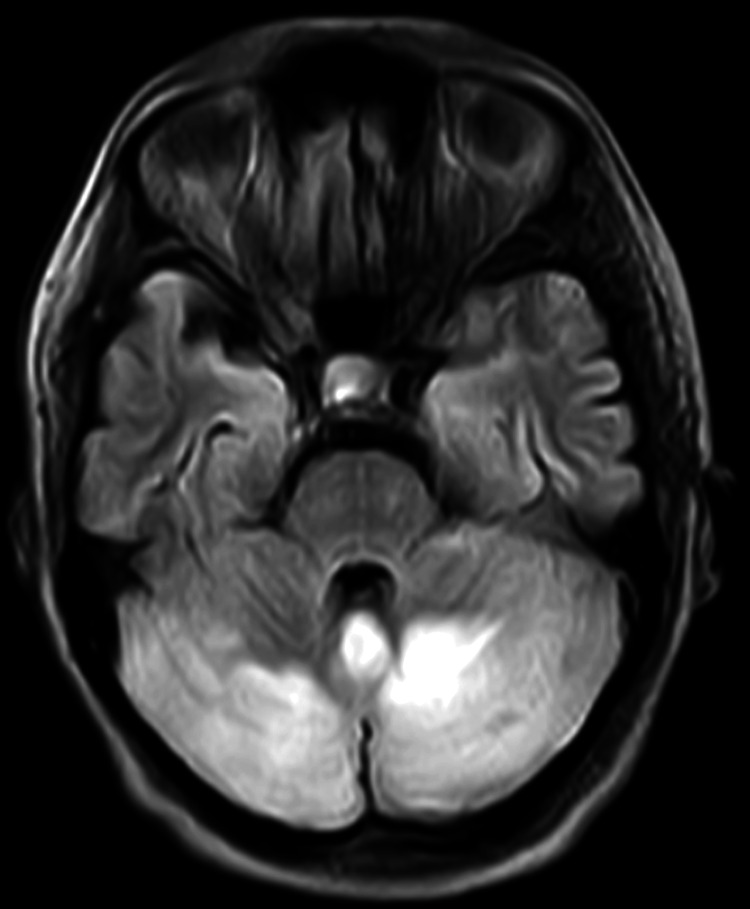
Diffusion restriction involving the cerebellum on MRI fluid-attenuated inversion recovery.

**Figure 2:**
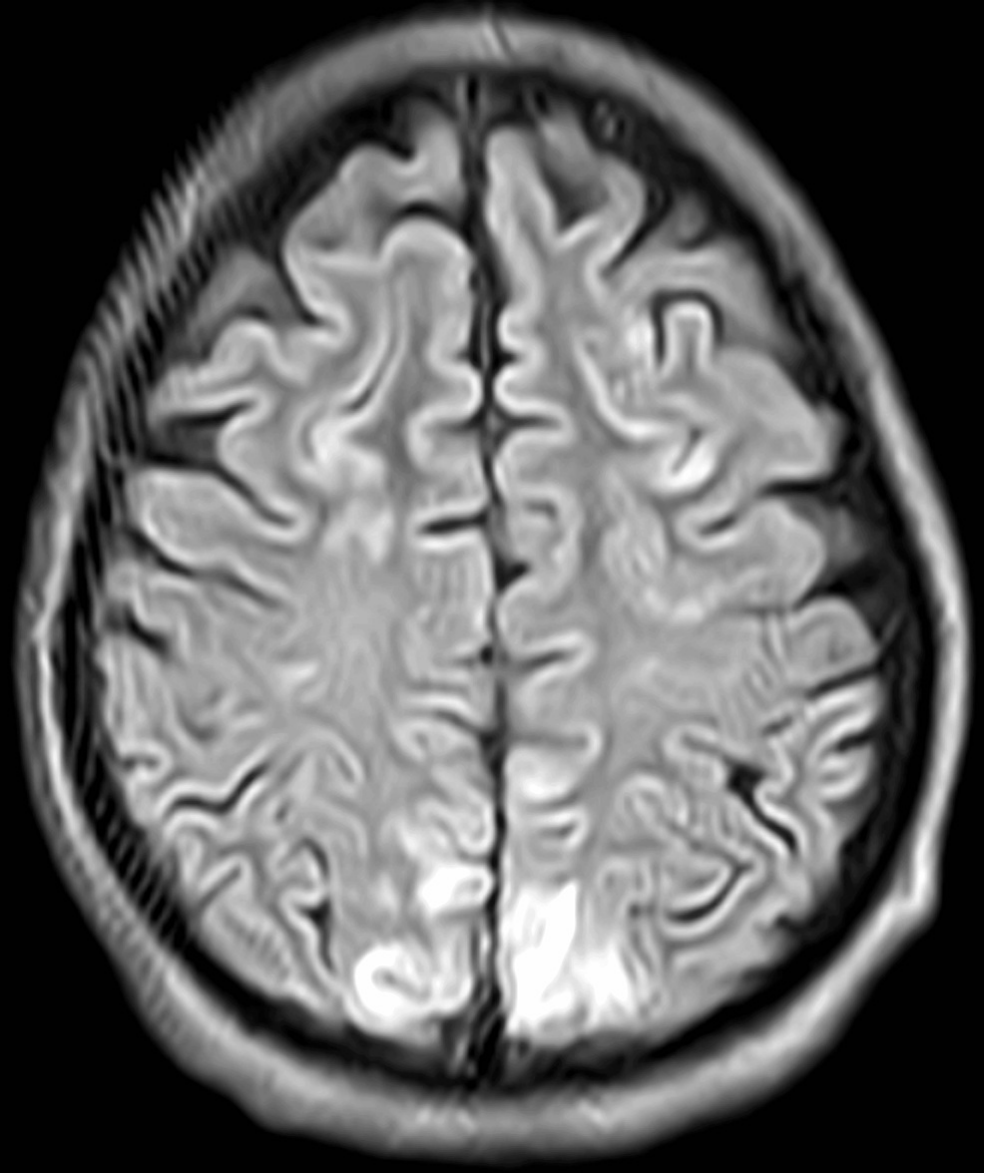
Diffusion restriction involving the occipital lobe on MRI fluid-attenuated inversion recovery.

**Figure 3:**
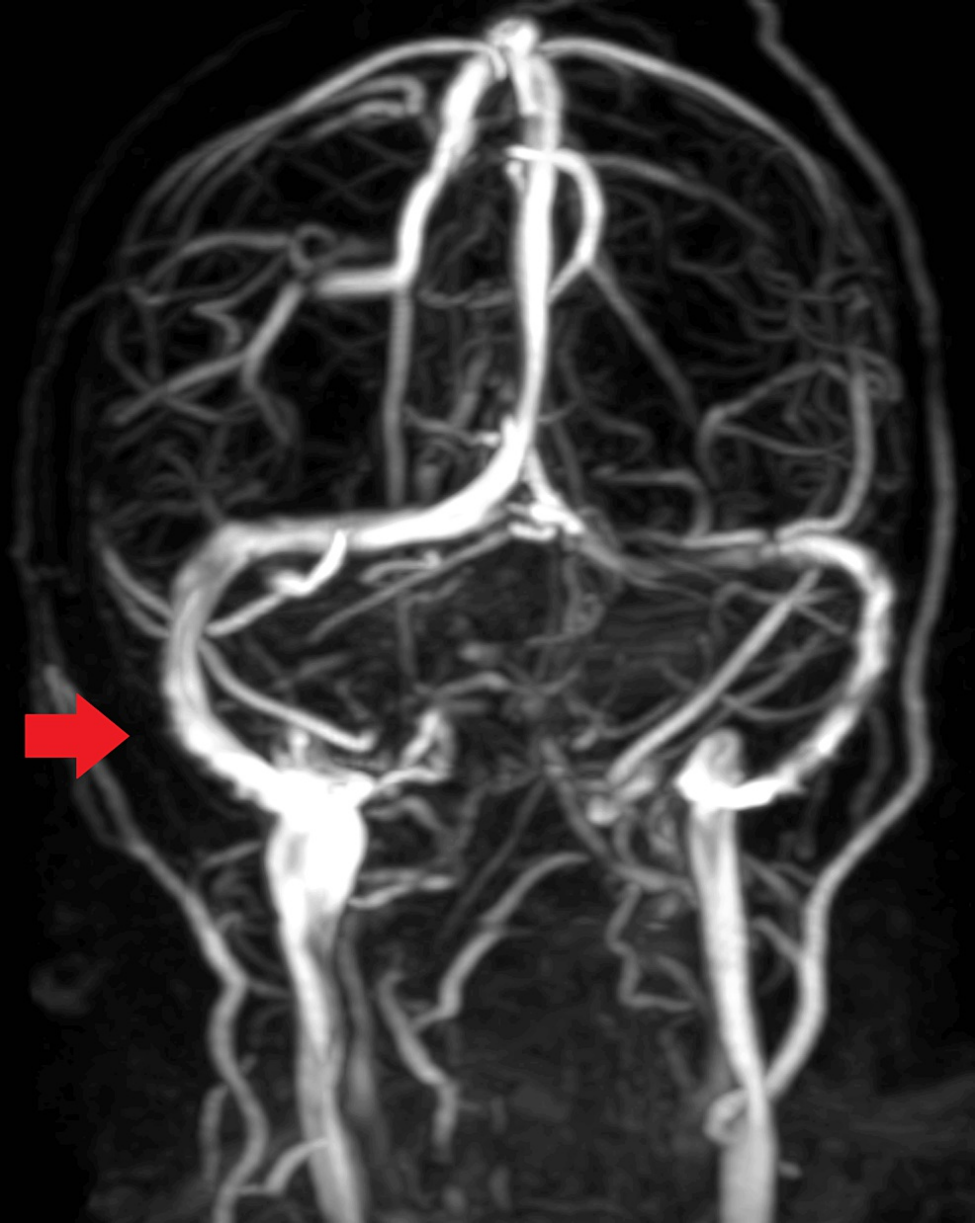
Narrowing of the transverse sinus seen on magnetic resonance venography.

Emergency laboratory testing revealed a lactate dehydrogenase level of 250 U/L, uric acid level of 3.8 mg/dL, 24-hour fluid intake of 3,150 mL, and 24-hour urine output of 2,700 mL. Urine analysis showed a protein-to-creatinine ratio of 3.7 g/gCr, and capillary blood glucose was low at 23 mg/dL. Anti-nuclear antibody counts were within normal limits at 1.34. Hemoglobin levels, coagulation panel, red blood cell count, renal function, and platelet count were normal. Fundus examination did not reveal signs of papilledema. MRI of the brain showed numerous regions of nearly symmetrical fluid-attenuated inversion recovery hyperintensities, with some areas showing diffusion restriction primarily involving the frontal, bilateral parieto-occipital, and both cerebellar lobes, indicative of PRES (Figures 1, 2). Narrowing of the transverse sinus was observed on magnetic resonance venography (Figure 3).

The patient was prescribed levetiracetam 500 mg twice daily for seven days, and her condition significantly improved from the second day post-seizure. She was discharged with instructions to continue levetiracetam 500 mg twice daily, along with calcium and iron supplementation. The patient was diagnosed with two conditions, namely, PRES and late postpartum eclampsia.

## Discussion

PRES is a rare postpartum diagnosis [7,8]. Patients typically present with non-specific neurological symptoms such as headaches and vision problems, which may progress to seizures or unconsciousness [2]. Eclampsia or pre-eclampsia are significant contributors to PRES in the obstetric population [5]. While these conditions often arise later in pregnancy, they can also occur postpartum. Abnormal placental development, possibly related to endothelial injury, is believed to play a role in PRES development in patients with pre-eclampsia and eclampsia [9]. Dysregulation of microRNAs (miRNAs), which govern biological processes such as cell division and growth, may contribute to aberrant placental vascularization, inflammation, and endothelial dysfunction [10]. Endothelial dysfunction, characterized by trans-endothelial leakage and exposure to free radicals, can lead to cerebral edema and compromise the motor or occipital cortex, resulting in eclamptic seizures or PRES manifestation, respectively [3].

Postpartum patients presenting with headaches, visual disturbances, edema, or seizures should be evaluated for PRES [9]. Our patient experienced PRES and eclampsia five days after delivery without prior specific pre-eclampsia symptoms. Notably, her blood pressure remained within normal limits during the postpartum period, and she had no history of pre-eclampsia or hypertension. This highlights the unpredictable nature of PRES and eclampsia [11,12]. MRI of the brain is the preferred imaging modality for diagnosing PRES. However, magnetic resonance venography and arteriography may be necessary to rule out other conditions, such as cerebral venous sinus thrombosis [4,10]. Treatment for PRES typically involves antihypertensive medication to lower blood pressure by 25% over 24 hours, along with seizure prophylaxis [12,13]. Antiepileptic medication can be gradually discontinued once the patient is asymptomatic and MRI lesions have resolved. Additionally, magnesium sulfate may be administered owing to its anticonvulsive and vasodilator properties [13].

Although patients with PRES generally have a favorable prognosis, they may still develop neurological complications such as cerebral ischemia and intracranial hemorrhage, including status epilepticus [1]. Hence, diagnosing and treating PRES to mitigate potential risks is crucial. Following treatment with magnesium sulfate, levetiracetam, and labetalol for one week after the eclamptic episode, our patient experienced complete recovery. During her follow-up appointment one month post-discharge, she reported no additional neurological issues, and a subsequent MRI three weeks later revealed no abnormalities. Given that PRES is associated with both maternal and fetal morbidity and mortality, early detection and prevention are paramount. Prediction of pre-eclampsia in women can be facilitated through the assessment of serum biomarkers such as soluble endoglin, natural killer cells, neurokinin B, miRNAs, endothelial progenitor cells, soluble fms-like tyrosine kinase 1 (sFlt-1), and placental growth factor (PlGF) [14]. Furthermore, the short-term absence of pre-eclampsia can be predicted in women using the sFlt-1/PlGF ratio [15]. Preventive measures, particularly for women at high risk of developing PRES and pre-eclampsia, may include the administration of low-dose aspirin between 11 and 36 weeks of gestation, as evidenced by the ASPRE trial [15]. However, further investigation is necessary to determine the efficacy and effectiveness of aspirin in preventing postpartum occurrences of these conditions.

## Conclusions

This case study underscores the importance of promptly diagnosing and treating PRES in the postpartum obstetric population. Patients presenting with symptoms such as headaches, seizures, and blurred vision during this phase should be evaluated for PRES and pre-eclampsia. The primary treatment modalities for PRES include magnesium sulfate, antihypertensive medications, and anticonvulsants. Additionally, aspirin has shown utility in preventing the development of both PRES and eclampsia. Further research is needed to deepen our understanding of the pathophysiology underlying PRES and eclampsia, as well as to explore additional preventive measures. This will contribute to improving outcomes and enhancing the management of these conditions in postpartum women.

## References

[1] Fischer M, Schmutzhard E (2017). Posterior reversible encephalopathy syndrome. J Neurol.

[2] Fugate JE, Rabinstein AA (2015). Posterior reversible encephalopathy syndrome: clinical and radiological manifestations, pathophysiology, and outstanding questions. Lancet Neurol.

[3] Staykov D, Schwab S (2012). Posterior reversible encephalopathy syndrome. J Intensive Care Med.

[4] McDermott M, Miller EC, Rundek T, Hurn PD, Bushnell CD (2018). Preeclampsia: association with posterior reversible encephalopathy syndrome and stroke. Stroke.

[5] Sibai BM, Stella CL (2009). Diagnosis and management of atypical preeclampsia-eclampsia. Am J Obstet Gynecol.

[6] Matthys LA, Coppage KH, Lambers DS, Barton JR, Sibai BM (2004). Delayed postpartum preeclampsia: an experience of 151 cases. Am J Obstet Gynecol.

[7] (2018). ACOG Committee Opinion No. 743: low-dose aspirin use during pregnancy. Obstet Gynecol.

[8] Laganà AS, Vitale SG, Sapia F (2018). miRNA expression for early diagnosis of preeclampsia onset: hope or hype?. J Matern Fetal Neonatal Med.

[9] Verhaegen J, Peeters F, Debois P, Jacquemyn Y (2019). Posterior reversible encephalopathy syndrome as a complication of pre-eclampsia in the early postpartum period. BMJ Case Rep.

[10] Hammer ES, Cipolla MJ (2015). Cerebrovascular dysfunction in preeclamptic pregnancies. Curr Hypertens Rep.

[11] Zhang L, Wang Y, Shi L, Cao J, Li Z, Wáng YX (2015). Late postpartum eclampsia complicated with posterior reversible encephalopathy syndrome: a case report and a literature review. Quant Imaging Med Surg.

[12] Lamy C, Oppenheim C, Mas JL (2014). Posterior reversible encephalopathy syndrome. Handb Clin Neurol.

[13] Zeisler H, Llurba E, Chantraine F (2016). Predictive value of the sFlt-1:PlGF ratio in women with suspected preeclampsia. N Engl J Med.

[14] Timofeeva AV, Gusar VA, Kan NE (2018). Identification of potential early biomarkers of preeclampsia. Placenta.

[15] Rolnik DL, Wright D, Poon LC (2017). ASPRE trial: performance of screening for preterm pre-eclampsia. Ultrasound Obstet Gynecol.

